# Editorial: ADAM, ADAMTS and astacin proteases: Challenges and breakthroughs in the -Omics era-Volume II

**DOI:** 10.3389/fmolb.2023.1172288

**Published:** 2023-04-07

**Authors:** Hang Fai Kwok, Kazuhiro Yamamoto, Rens de Groot, Simone Dario Scilabra, Salvatore Santamaria

**Affiliations:** ^1^ Cancer Centre, Faculty of Health Sciences, MOE Frontiers Science Center for Precision Oncology, University of Macau, Avenida de Universidade, Taipa, China; ^2^ Institute of Life Course and Medical Sciences, University of Liverpool, Liverpool, United Kingdom; ^3^ Institute of Cardiovascular Science, University College London, London, United Kingdom; ^4^ Proteomics Group of Fondazione Ri.MED, Department of Research IRCCS ISMETT, Palermo, Italy; ^5^ Department of Biochemical Sciences, School of Biosciences, Faculty of Health and Medical Sciences, University of Surrey, Guildford, United Kingdom

**Keywords:** ADAM, ADAMTS, astacin, extracellular matrix, proteases, reprolysin family of zinc metalloproteases, M12B metalloproteases

ADAMs (A Disintegrin and Metalloproteinases), ADAMTS (A Disintegrin and Metalloproteinase with Thrombospondin motifs) and astacins are three sub-families of proteases belonging to the metzincin superfamily. In Volume II of ADAM, ADAMTS and Astacin Proteases: Challenges and Breakthroughs in the -Omics Era we illustrate how the -Omics Era has changed our comprehension of the biological roles of these proteases.

An analysis of representative molecular structures, sequence similarity and phylogenetic origins of astacins performed by Gomis-Rüth and Stöcker identified the potential origin of astacins in unicellular holozoans, the precursor of metazoans (multicellular organisms). They suggest that the scattered presence of astacins across the different kingdoms of life may have occurred due to horizontal gene transfer from holozoans.

Meprin β is a type I transmembrane astacin the expression of which is associated with certain types of cancer, including melanoma. Volume I of this Research Topic showed the impact of meprin β variants on the invasiveness of tumor cells (Gellrich et al., 2021). In this Volume II, Wöhner et al. identified meprin β as a protease responsible for ectodomain shedding of CD44, a transmembrane glycoprotein expressed in several different tumors. Proteolytic processing of the CD44 ectodomain leads to release of intracellular fragments, which triggers transcriptional modifications. The authors found a unique regulatory loop at the transcriptional level for CD44, matrix metalloproteinase 2 (MMP2) and CD44 sheddases including MMP14 and ADAM10, which may contribute to the development of cancer.

ADAM17 is another metzincin that has attracted considerable interest due to its involvement in inflammation and cancer (Moss and Minond, 2017). A novel bi-specific inhibitor was developed by Weizman et al. that simultaneously targets ADAM17 and the proinflammatory cytokine TL1A. They propose it has high therapeutic potential in the treatment of inflammatory bowel disease. When fused together, the two inhibitory domains displayed a synergistic effect that dramatically increased the potency of this novel therapeutic option.

The ADAMTS sub-family of metzincins comprises 19 secreted members in humans, with roles ranging from cleavage of extracellular matrix (ECM) proteins to platelet aggregation (Dubail and Apte, 2015). ADAMTS5 is best known for its role in osteoarthritis, where it drives cartilage degradation by cleaving the proteoglycan aggrecan (Santamaria, 2020). A single nucleotide polymorphism in *ADAMTS5* has been associated with severe lumbar disc degeneration (Rajasekaran et al., 2015) and *Adamts5* deletion protected from intervertebral disc degeneration (IDD) in a mouse model of the disease (Ngo et al., 2017). Jing and Liu describe an interplay between long nuclear RNAs (lncRNAs) and microRNAs (miRNAs) in the regulation of *ADAMTS5* expression in ID-derived nucleus pulpous cells. The lncRNA HOXC13-antisense (AS) induced a catabolic response characterized by increased expression of ADAMTS5 as well as MMP3, ADAMTS4 and a number of pro-inflammatory cytokines, while down-regulating expression of anabolic markers such as aggrecan and collagen type II. This action of lncRNA HOXC13-AS was directly antagonized by miR-497-5p, which down-regulated *ADAMTS5* expression and whose expression levels negatively correlated with IDD severity in patients. Since HOXC13-AS down-regulated miR-497-5p expression, this lncRNA may represent a master regulator of ADAMTS5 in intervertebral discs. Although other microRNAs have been involved in ADAMTS5 regulation such as miR-137 (Zhang et al., 2019) and miR-145 (Hu et al., 2017), expression of ADAMTS5 is also regulated at the post-translational level (Santamaria, 2020), therefore dysregulated ADAMTS5 activity may be a product of multiple converging regulatory mechanisms.

Null mutations in *ADAMTS10* are associated with a recessive form of Weill-Marchesani syndrome, characterized by eye abnormalities (Dagoneau et al., 2004). Mutations in fibrillin-1 (*FBN1*) lead to a dominant, albeit clinically indistinguishable, form of the syndrome (Faivre et al., 2003), indicating overlapping functions of the two proteins. Fibrillin1 microfibrils can function as reservoirs of latent transforming growth factor beta (TGFβ), thereby controlling TGFβ availability and function. Wareham et al. used zebrafish (*Danio rerio*) as an *in vivo* model to investigate the link between ADAMTS10 and fibrillin1 in ocular development. They found that ADAMTS10 is required to cleave fibrillin1 microfibrils and liberate TGFβ. In turn, activation of the TGFβ pathway is necessary for the development of the optic nerve. Altogether, the study reported a novel function of ADAMTS10 in the development of retinal ganglion cells, that is mediated by its ability to liberate TGFβ from fibrillin1 microfibrils and initiate TGFβ signaling.

The link between TGFβ pathway, fibrillin microfibrils and ADAMTSs is further explored in the review paper by Mead on ADAMTS6, a protease involved in musculoskeletal and cardiovascular development (Santamaria and de Groot, 2020). Deletion of *Adamts6* in mice is embryonically lethal due to various congenital heart defects (Prins et al., 2018). Previous work by Mead et al. (2022) has shown that the knockout embryos also present with appendicular skeletal abnormalities as well as axial skeleton manifestations and facial deformities. The molecular mechanisms are still being elucidated but ADAMTS6 binding to and cleavage of both fibrillins and latent TGFβ binding proteins (LTBPs) point to a role in TGFβ signaling (Cain et al., 2022; Mead et al., 2022). Indeed, a recent study by Cain et al. (2022) showed that ADAMTS6 can cleave LTBP3 and overexpression of ADAMTS6 increased TGFβ activation in a dose dependent manner, following stimulation with mature TGFβ1. ADAMTS6 was first identified to be expressed in human placental tissue (Hurskainen et al., 1999) and the Human Protein Atlas reports that in humans ADAMTS6 is expressed in the placenta and endometrium at both RNA and protein levels (https://www.proteinatlas.org/ENSG00000049192-ADAMTS6/tissue). The Cancer Genome Atlas also shows that both ADAMTS6 and fibrillin3 are expressed in endometrial cancer, the latter being an unfavorable prognostic marker. Wächter et al. (2022) found co-expression of ADAMTS6 and fibrillin-2 in cytotrophoblasts and, in the ClinVar database, an *ADAMTS6* mutation in a patient with primary ovarian insufficiency has been reported as likely pathogenic. Together, these data suggest a role of ADAMTS6 in pregnancy and possibly in endometrial cancer, which also needs to be further explored.

In summary, this Volume II elucidates novel roles of ADAMs, ADAMTSs and astacins in development, inflammatory and degenerative conditions and cancer. It also offers innovative approaches to target dysregulated metzincin activities at different levels of regulation. The picture that emerges from these studies is one characterized by many interconnections between ECM proteins and exquisite fine tuning of proteolytic activity. As highlighted in this Research Topic, tackling such a daunting complexity requires a multidisciplinary as well as mechanistic approach.
